# Erratum: Environmental change and long-term body mass declines in an alpine mammal

**DOI:** 10.1186/s12983-014-0088-3

**Published:** 2014-12-05

**Authors:** Tom HE Mason, Marco Apollonio, Roberta Chirichella, Stephen G Willis, Philip A Stephens

**Affiliations:** School of Biological and Biomedical Sciences, Durham University, South Road, Durham, DH1 3LE UK; Department of Science for Nature and Environmental Resources, University of Sassari, via Muroni 25, Sassari, I-07100 Sardinia Italy

## Erratum

There is an inconsistency between the sample size quoted in the Methods section and that presented in Additional file 1 [1]. The number quoted in the Methods of 10,455 individuals (5,762 males and 4,693 females) is incorrect. This figure relates to our full database, which includes some individuals shot beyond the boundaries of the areas focused on in this study. Because those individuals came from areas with limited representation in the database, they were excluded from our analysis. Following their exclusion, our sample size was 9,388 individuals (5,218 males and 4,170 females, as displayed in Additional file 1); this is the number that should have been quoted in the main text. Importantly, since the excluded data were removed before we conducted our analysis, our results and conclusions are unchanged. We apologise for any potential confusion this may have caused.
